# Letter to the editor: Atypical age distribution and high disease severity in children with RSV infections during two irregular epidemic seasons throughout the COVID-19 pandemic

**DOI:** 10.2807/1560-7917.ES.2024.29.20.2400269

**Published:** 2024-05-16

**Authors:** Xiangting Liu, Guangting Zeng

**Affiliations:** 1 Reproductive Medicine Center, The First People’s Hospital of Chenzhou, Xiangnan University, Chenzhou, China; 2 Department of Pharmacy, The First People's Hospital of Chenzhou, Xiangnan University, Chenzhou, China

**Keywords:** RSV, children, COVID-19

**To the editor:** Respiratory syncytial virus (RSV) showed different epidemiological patterns before and after the COVID-19 pandemic. A post-pandemic surge in cases has been observed in some countries [1]. The reasons and mechanisms behind this phenomenon remain unclear. Several hypotheses have been proposed, including a decline in immunity against RSV due to prolonged lack of exposure to this virus, also known as the immune debt hypothesis, immune impairment caused by COVID-19, changes in testing policies and healthcare seeking behaviours, and the emergence of new RSV lineages [2].

Cai et al. described the 2021 and 2022/23 RSV seasons in children < 5 years of age in Germany [3]. They suggest that the surge in the number and severity of RSV infections in children may be due to a lack of post-RSV exposure resulting in lower population immunity. Here we highlight several points of this study worth discussing. First, we would like to recognise the work of the authors, who performed genetic sequencing analysis of the circulating strains. Analysis of the viral genome showed that circulating strains between 2021 and 2023 remained of the same RSV genotypes as in previous seasons, which suggests that viral factors are unlikely to be responsible for the resulting altered epidemiologic patterns.

Second, the immune debt hypothesis is widely accepted, including by the authors. Interestingly, during the COVID-19 pandemic the RSV positivity rate of both RSV seasons was significantly higher compared with pre-COVID-19 RSV seasons. However, compared with pre-COVID-19 RSV seasons, in the 2021 season, the proportion of RSV cases admitted to the intensive care unit (ICU) among hospitalised RSV cases was reduced, while the proportion needing ventilator support remained unchanged. In the 2022/23 season, a significantly higher proportion of RSV cases aged < 5 years was admitted to ICU and required ventilator support compared with pre-COVID-19 RSV seasons. In particular, in infants aged < 3 months the proportion needing mechanical ventilation was significantly increased. These observations do not seem to be explained by the immune debt hypothesis, since exposure restrictions were lifted in 2021. It seems unlikely that infants aged 0–3 months in the years 2022 and 2023 would have an immune debt.

As for the immune injury hypothesis caused by COVID-19, the effect of severe acute respiratory syndrome coronavirus 2 (SARS-CoV-2) infection cannot be determined because this study did not consider SARS-CoV-2 infection in children. Current studies have revealed that SARS-CoV-2 infection may cause immune injury in children and increase their susceptibility to other respiratory viruses, such as RSV [4]. The mechanisms of immune injury include a decrease in the proportion of cluster of differentiation (CD)4 and CD8 T-cells and an increase in the expression of markers of exhausted T-cells and B-cells after infection [5], and this immune injury may persist for several months. More comprehensive and robust studies are needed to examine the clinical outcomes of RSV infection in patients with and without previous SARS-CoV-2 infection.

In addition, this study did not look at changes in testing before and after the pandemic. As a result of the COVID-19 pandemic, the detection capacity for respiratory pathogens has increased. Screening for SARS-CoV-2 is often accompanied by testing for other pathogens. This means that the RSV testing rate is likely to grow substantially as well. A study by Petros et al. suggests that the increase in RSV in children after the emergence of COVID-19 can be attributed to an increase in testing [6].

In summary, the reasons for the changes in the epidemiological pattern of RSV before and after COVID-19 are complex and unclear. More research is needed to understand drivers of respiratory virus epidemiology, as this knowledge can help guide RSV prevention and healthcare resource planning.
